# Identification of PMD subgroups using a myelination score for PMD

**DOI:** 10.1016/j.ejpn.2022.10.003

**Published:** 2022-11-04

**Authors:** Inga Harting, Sven F. Garbade, Stefan D. Rosendaal, Alexander Mohr, Omar Sherbini, Adeline Vanderver, Nicole I. Wolf

**Affiliations:** a Department of Neuroradiology, University Hospital Heidelberg, Im Neuenheimer Feld 400, 60120, Heidelberg, Germany; b Centre for Child and Adolescent Medicine, Clinic I, Division of Child Neurology and Metabolic Medicine, University Hospital Heidelberg, Im Neuenheimer Feld 669, 69120, Heidelberg, Germany; c Department of Radiology, Amsterdam UMC, Amsterdam, the Netherlands; d Division of Neurology, Department of Pediatrics, Children’s Hospital of Philadelphia, Philadelphia, PA, USA; e Department of Neurology, Perelman School of Medicine, University of Pennsylvania, Philadelphia, PA, USA; f Department of Child Neurology, Amsterdam Leukodystrophy Center, Emma Children’s Hospital, Amsterdam University Medical Centers, VU University, and Amsterdam Neuroscience, Cellular&Molecular Mechanisms, Amsterdam, the Netherlands

**Keywords:** PLP1, MRI, Hypomyelination, Myelination, Score, Pelizaeus-Merzbacher disease

## Abstract

**Background:**

The clinical spectrum of Pelizaeus-Merzbacher disease (PMD), a common hypomyelinating leukodystrophy, ranges between severe neonatal onset and a relatively stable presentation with later onset and mainly lower limb spasticity. In view of emerging treatment options and in order to grade severity and progression, we developed a PMD myelination score.

**Methods:**

Myelination was scored in 15 anatomic sites (items) on conventional T2-and T1w images in controls (n = 328) and 28 PMD patients (53 MRI; n = 5 connatal, n = 3 transitional, n = 10 classic, n = 3 intermediate, n = 2 PLP0, n = 3 SPG2, n = 2 female). Items included in the score were selected based on interrater variability, practicability of scoring and importance of scoring items for discrimination between patients and controls and between patient subgroups. Bicaudate ratio, maximal sagittal pons diameter, and visual assessment of midsagittal corpus callosum were separately recorded.

**Results:**

The resulting myelination score consisting of 8 T2-and 5 T1-items differentiates patients and controls as well as patient subgroups at first MRI. There was very little myelin and early loss in severely affected connatal and transitional patients, more, though still severely deficient myelin in classic PMD, ongoing myelination during childhood in classic and intermediate PMD. Atrophy, present in 50% of patients, increased with age at imaging.

**Conclusions:**

The proposed myelination score allows stratification of PMD patients and standardized assessment of follow-up. Loss of myelin in severely affected and PLP0 patients and progressing myelination in classic and intermediate PMD must be considered when evaluating treatment efficacy.

## 1. Introduction

Pelizaeus-Merzbacher disease (PMD, OMIM #312080) is one of the most common hypomyelinating leukodystrophies, a group of genetic white matter disorders defined by a significant and permanent deficit in CNS myelin [1,2]. PMD is caused by variants in the x-chromosomal gene coding the most abundant myelin protein, proteolipid protein 1 (PLP1), including duplications (60–70%), point mutations (~20%), and infrequent deletions [3–9]. Advances in clinical genetic testing have revealed a wider spectrum of clinical presentation than initially thought, ranging from a severe, neonatal presentation to pauci-symptomatic adults. Clinically, PMD is classified into multiple subtypes: Connatal PMD is characterized by neonatal onset with nystagmus, hypotonia, stridor, and evolving spasticity, ataxia, and extrapyramidal hyperkinesia. Transitional PMD has its onset in the neonatal or early infantile period, but without connatal stridor. Its course is less rapid than in connatal type PMD, and it shares features of classic PMD, albeit head control is not achieved. Classic PMD, the most common type, manifests in the first year of life with nystagmus, reduced muscle tone of the legs, head titubation, and muscular hypotonia, with limited gains in developmental milestones and development of spasticity. Further, less severe phenotypes include intermediate PMD with patients achieving independent sitting as best motor function, pure and complicated spastic paraplegia type 2 (SPG2), the latter combining features of SGP2 and PMD with early onset and progression. While closely overlapping with intermediate PMD and spastic paraplegia, *PLP1* null syndrome with no protein product (PLP0) is set apart by the presence of mild peripheral neuropathy. Female carriers are commonly clinically normal, and only in rare instances show symptoms in infancy and early childhood, often transient. In adulthood, female carriers may manifest with neurological signs, e.g., mild spastic paraparesis, and an inverse relationship between severity in affected males and likelihood of manifestation in heterozygote females has been noted in some families [8,10–14].

While there is no simple genotype-phenotype-correlation as a basis for individual prognosis, different types of genetic variants are associated with differently severe manifestations. Duplications commonly cause classic PMD. Missense variants are found throughout the entire gene and may lead to the entire PMD spectrum. Loss of function-variants (also called PLP null syndrome) lead to an initially less severe presentation, with subsequent clinical deterioration [8,14,15].

Imaging-wise, lack of myelin is the hallmark of PMD. Lack of myelin has been shown to at least codetermine motor handicap in patients with hypomyelinating leukodystrophies [1,16] and functional disability in PMD correlates with white matter volume and degree of hypomyelination [17–19]. In view of emerging therapeutic approaches, identification of distinct clinical cohorts and biomarkers for grading of severity and progression is crucial. To this end we developed an MRI severity score assessing the degree of myelin deficit and atrophy using conventional T2-and T1-weighted images, which are available for most patients and can thus be drawn on for both retrospective studies of natural history and routine clinical use.

## 2. Patients and methods

### 2.1. Patient selection

For development of the MRI score and analysis of temporal changes patients with genetically proven Pelizaeus-Merzbacher disease imaged up to the age of 20 years were retrospectively identified using the database of VU Medical Center in Amsterdam. MRI scans had been acquired at different scanners (1.5 and 3T) using different protocols and were only included if axial T2-and T1-weighted images and a sagittal sequence were available. Patients were clinically assessed as belonging to specific subtypes of the PMD spectrum by a paediatric neurologist with long-time experience in hypomyelinating disorders (NIW).

For interrater reliability six MRI scans of five genetically proven PMD patients were collected from the Myelin Disorders Bioregistry Project at the Children’s Hospital of Philadelphia.

### 2.2. Controls

**Fig. 1.**
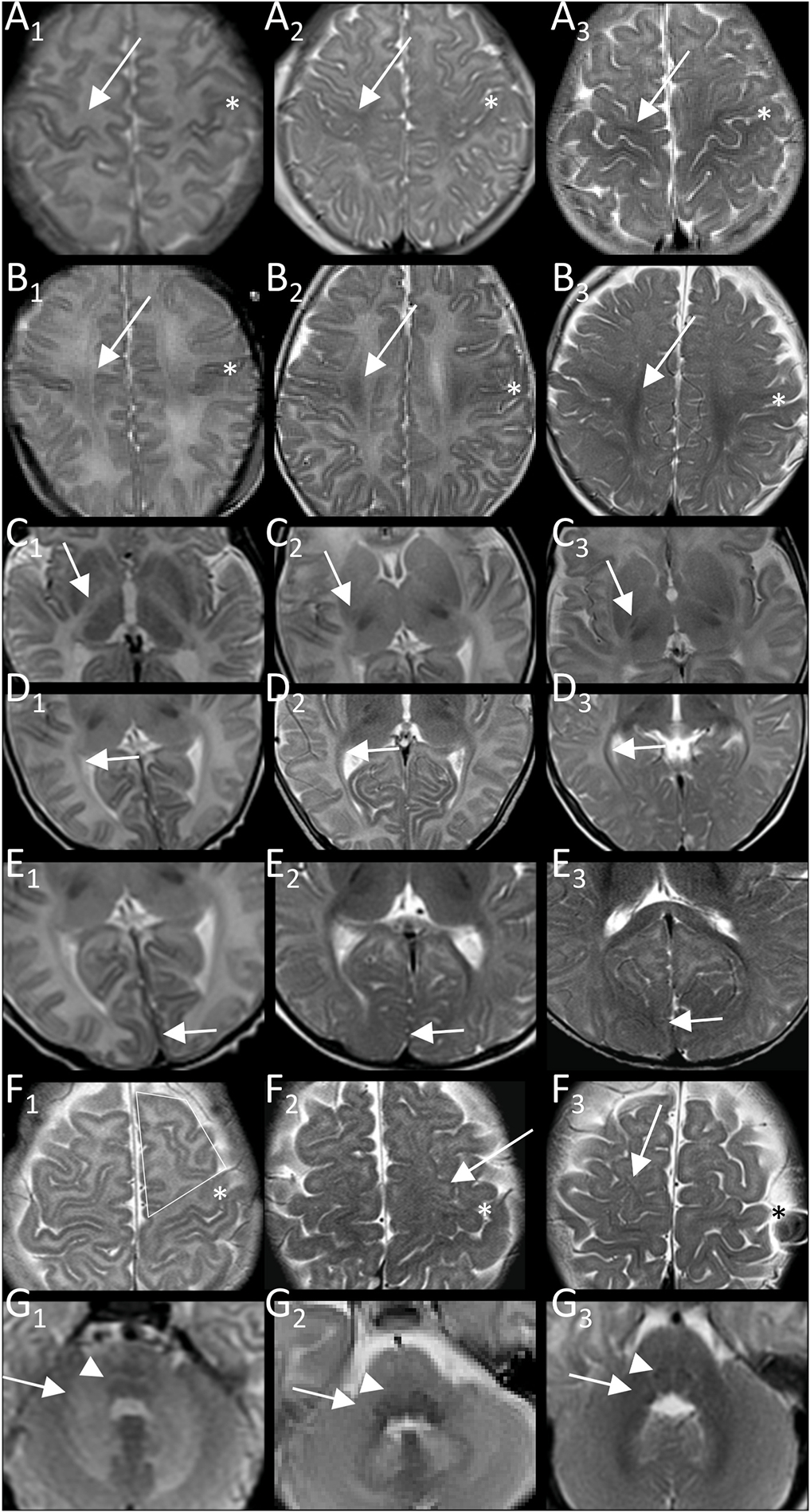
Examples of T2-item scoring. Best myelination of pyramidal tract in central region (A), centrum semiovale (B), and PLIC (C), of visual tract in peri-trigonal optic radiation (D) and primary visual region (E), of subcortical frontal not central white matter (F) and middle cerebellar peduncle (G; at origin in pons) with T2-hyper/iso/hypointensity relative to cortex corresponding to values of 0, 1, 2 in column A_1_-G_1_, A_2_-G_2_, and A_3_-G_3_, respectively. More T2-hypointense cortex of central region is reference for central region and centrum semiovale (*central sulcus). Medial lemniscus is scored as abnormally T2-hyperintense (value 0, G_1_, image of connatal patient as not seen in controls) versus normally T2-iso/hypointense (G_2,3_, value 1). Arrows indicate area scored.

Normal myelination was retrospectively assessed in 374 MRI scans of 364 patients imaged between 0 and 20 years with normal findings on cranial MRI, excluding patients with significant developmental delay, known epilepsy, and a history of intraventricular or subarachnoid haemorrhage (163 female, 211 male, mean age at MRI 3.13, median 1.31 years). Sampling density was age-adapted in order to account for the time frame of myelination (Suppl.Figure1).

### 2.3. MRI scoring

Myelination of patients and controls was graded separately on T2- and T1-weighted images (T2w, T1w) as best myelination in a total of 15 specific anatomic regions (“items”) by an experienced paediatric neuroradiologist blinded to the clinical diagnosis (IH). Scoring items represent supratentorial primary motor and visual pathways, early myelinating infratentorial structures, as well as later myelinating supratentorial white matter regions (Suppl.Table1). 13 of these 15 items were scored depending on their signal in relation to cortex, while T2-signal of pyramidal tract and medial lemniscus in pons was scored relative to signal of surrounding brainstem white matter.

The validity of the items as a model of myelination was tested by performing principal component analysis (PCA) for T2-and T1-items in controls. As the first principal component explained 85.0% (T2w) and 85.4% (T1w) of the variation of the model and only the first principal component had an eigenvalue greater 1, an unifactorial model was thus sufficient to explain the variance of all items. This is consistent with the hypothesis that changes of the items as surrogate parameters of myelination are primarily dependent on age with only minor influences due to differences in imaging sequences and reading of MRIs.

For investigation of inter-rater reliability two further experienced pediatric neuroradiologists (AM, SR) were trained by the first, before all three raters scored six MRI scans of five new PMD patients. Concordance among raters for ordinal data was computed using linear weights in order not to be affected by kappa paradoxes, in particular by restricted range lowering the estimates of inter-rater reliability [20]. The three T2-items ALIC (0.5), pyramidal tract in pons, and simplified scoring of genu (0.56) had only “fair concordance” between inter-raters and were discarded while, while except for T2-scoring of peridentate white matter (0.62), concordance of all other items was excellent ( ≥ 0.75) and items retained. Importance of scoring items for discrimination between PMD patients and controls and between PMD subgroups was investigated visually and using curves of receiver operation characteristics (ROC).

### 2.4. Surrogate parameters of brain volume

**Fig. 2.**
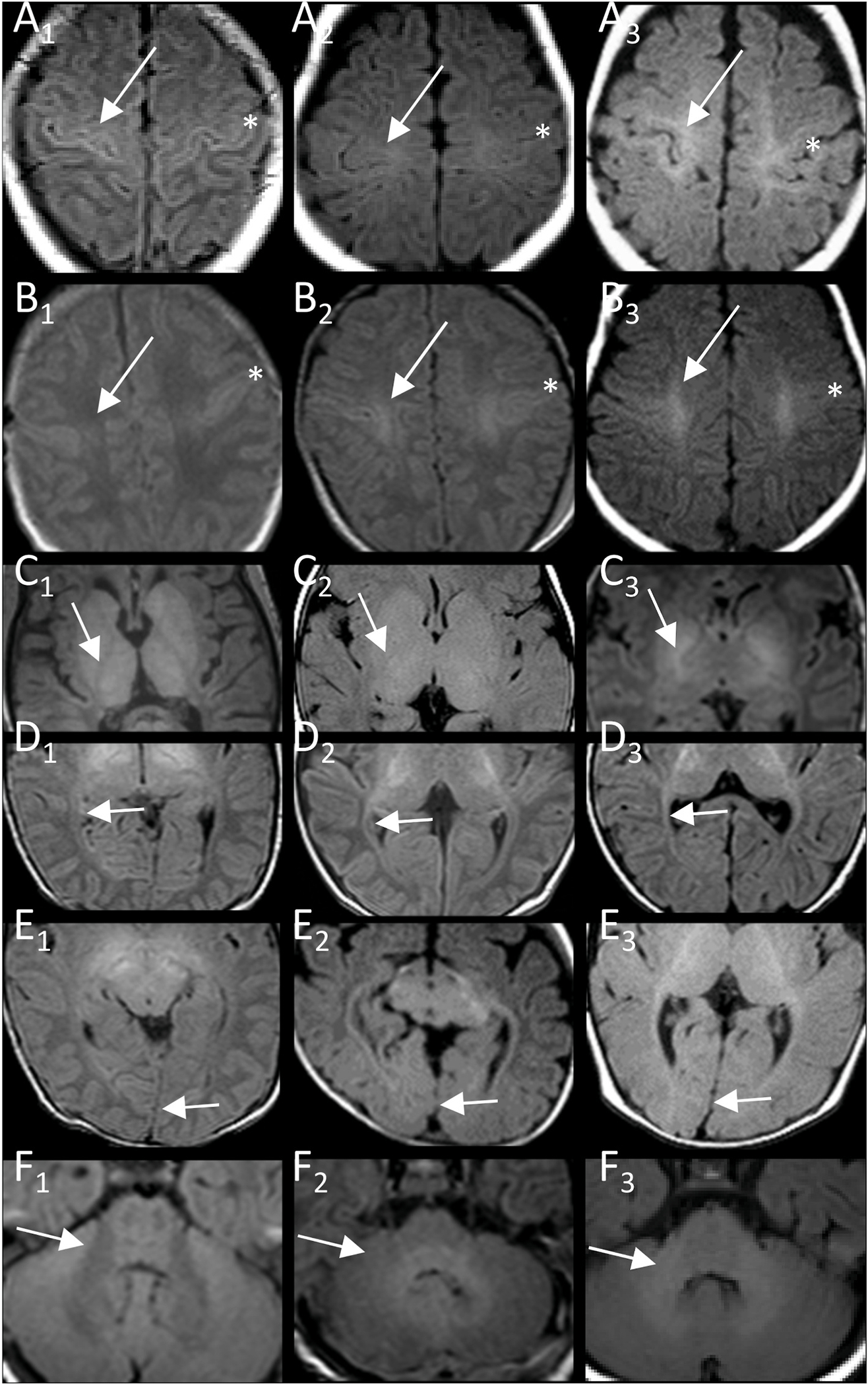
Examples of T1-item scoring. Best myelination of pyramidal tract in central region (A), centrum semiovale (B), and PLIC (C), of visual tract in optic radiation (D, peritrigonal) and primary visual region (E), and of middle cerebellar peduncle (F) with T1-hypo/iso/hyperintensity relative to cortex corresponding to values of 0, 1, 2 in column A_1_-F_1_, A_2_-F_2_, and A_3_-F_3_, respectively. More T1-hyperintense cortex of central region is reference for central region and centrum semiovale (*central sulcus). Arrows indicate area scored.

The bicaudate ratio (BCR), visual scoring of the corpus callosum on midsagittal images (thin y/n, e.g. Suppl.Figure 2), and the maximum anterior-posterior diameter of the pons on sagittal images were used as surrogate parameters of brain volume. BCR, defined as the minimum intercaudate distance divided by the transverse width of the inner table of the skull at the same level, i.e., the outer surface of CSF signal, was measured using axial T2-weighted images where the caudate heads were most visible and closest to one another. Raw values of BCR and pons diameter were compared with controls using z-scores and age-adapted controls [21].

### 2.5. Statistical analysis

Statistical analysis was performed using R environment for statistical computing and graphics (R, 2020). Principal component analysis (PCA) for validation of T2-and T1-scoring items as a model of myelination was implemented through R function *prcomp* [22], analysis of receiver operating characteristics (ROC) of differently composed scores through R package *pROC* [23]. Concordance among inter-raters using linear weights was implemented through R package *raters* Version 2.0.1, cut-offs were derived from Cicchetti, values less than 0.40 being considered as poor and ratings between 0.40 and 0.59 categorized as fair, between 0.60 and 0.74 as good, and between 0.75 and 1.00 as excellent [24]. Scores of subgroups were compared using the pairwise Wilcoxon rank sum-test with Holm adjustment for multiple testing as non-parametric test through R package *rstatix* [25].

## 3. Results

### 3.1. Patients

28 PMD patients first imaged at 0.15–11.95 years (mean 2.85, median 1.30 years) were identified for score development and a total of 53 brain MR scans including 25 follow-up scans in 19 patients for assessment of longitudinal changes were collected (0.15–14.42 years, mean 4.07, median 2.18). The largest subgroup were patients with classic PMD (n = 10) followed by patients with congenital PMD (n = 5), three patients each with transitional, intermediate PMD, and SPG2, two patients with PLP0-mutations and two female patients (Suppl.Table2). Myelination was deficient for age in all patients and MRIs.

### 3.2. PMD myelination score

For score development only the first MRI scans were used as delineation of deficient myelination is more difficult at younger ages. Patients with mild hypomyelination and/or first MRI in adolescence were not excluded, as they are the most difficult ones to distinguish from controls. Items were retained or discarded based on practicability of scoring and importance for discrimination between patients and controls as well as between subgroups of PMD patients (Suppl. Material: Selection of T2- and T1-items for the myelination score).

The resulting score included eight T2-and six T1-items combining the five T2-and T1-items of supratentorial pyramidal and visual tract (central region, centrum semiovale, PLIC, optic radiation, and primary visual area) with the T2-items of subcortical frontal white matter, medial lemniscus, and MCP and the T1-item of MCP (Table 1, Figs. 1 and 2) While a basic T2-score of supratentorial pyramidal and visual tract items was sufficient for discrimination of patients and controls, the three additional T2-items increased differentiation of subgroups and as did the T1w-items, which were not primarily suited for differentiating patients and controls.

**Fig. 3.**
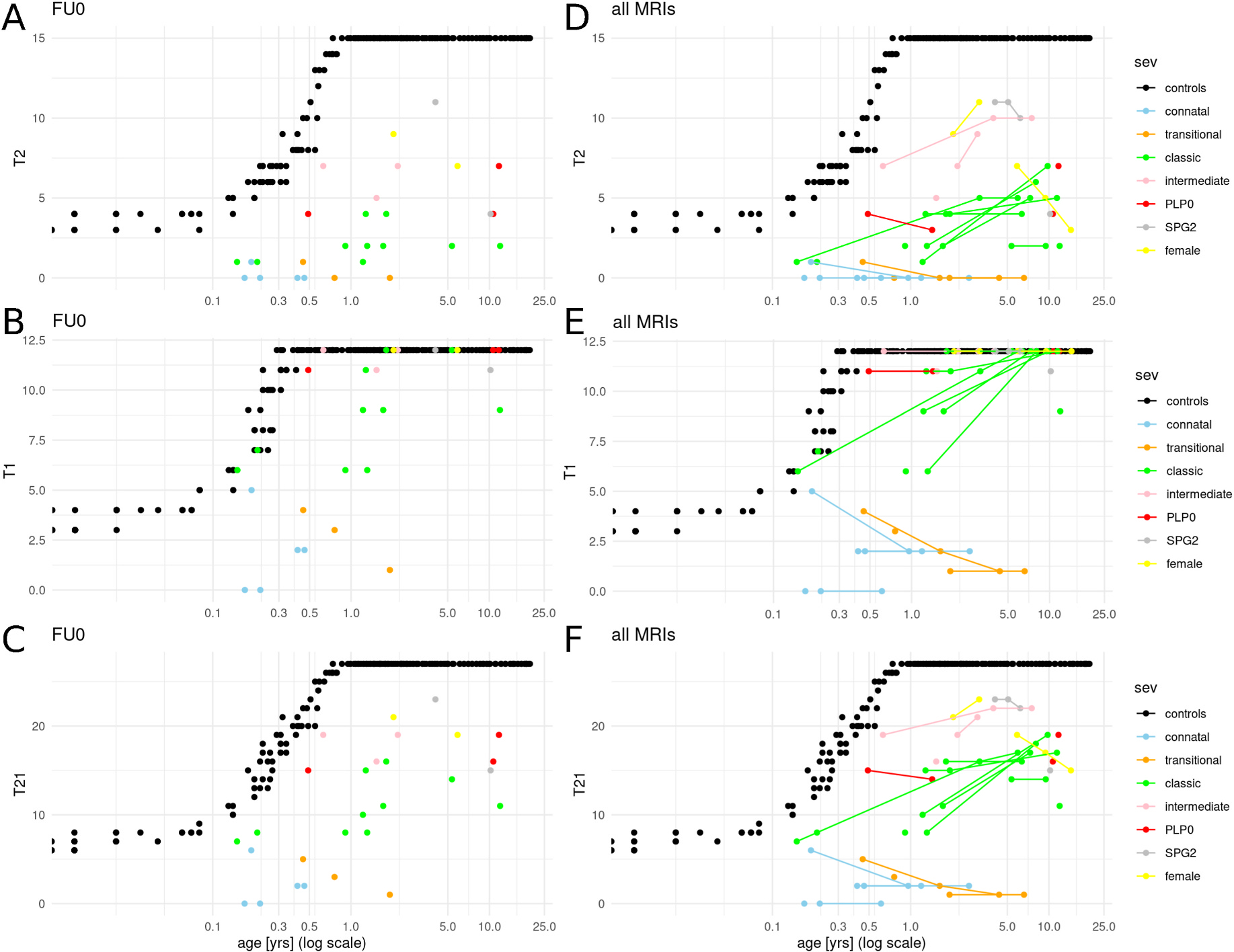
T2-, T1-and combined T21-, as well as combined T21-myelination and brain volume score for first and all MRIs of PMD patients. The final T21score (C, F) combines T2-based distinction between patients and controls (A, D) with subgroup differentiation with larger contribution of T1-items (B, E). Score were developed based on scoring of first MRI (FU0; A-C). Inclusion of follow-up MRIs (D-F; lines connecting scores of individual patients at different time points) visualizes the diverging natural history of subgroups with increasing myelination score predominantly in patients with classic PMD in contrast to stable or decreasing scores in connatal and transitional patients. (controls: black; connatal: light blue; transitional: orange; classic: green; intermediate: pink; PLP0: red, SPG2: grey; female: yellow; logarithmic x-axis with age in years.).

The resulting myelination score consisting of eight T2-and six T1-items allowed discrimination between patient and controls as well as classic and connatal patients when plotted (Fig. 3C). On ROC analysis, overlapping score values of better myelinated PMD patients with those from younger, not fully myelinated controls resulted in an AUC 89.2%. While there was no overlap between patients with connatal and transitional vs. classic PMD (AUC 100%, cut-off score 6.5), overlapping scores of patients with classic PMD and those with intermediate PMD or PLP0 precluded differentiation of these subgroups based on MRI alone (AUC 96%, cut-off score 14.5, Suppl.Figure 3C).

### 3.3. Myelination in different types of PMD at first MRI

Patients with connatal PMD had earliest imaging at 0.17–0.46 years and lowest mean T2-, T1-, and T21-myelination scores. Individual scores overlapped with those of transitional PMD patients who had slightly higher mean scores (Suppl.Table3, Fig. 3C). All T2-items were hyperintense with the exception of transient T2-isointese myelin in the early acquired first MRI scans of one connatal (0.19 years, PLIC, lost by 0.96 years) and one transitional patient (0.45 years, optic radiation, lost by 1.62 years). Of T1-items, MCP was isointense in three connatal and all transitional patients, PLIC at least T1-isointense in three of five connatal and two of three transitional patients, and the same two early imaged patients also lost their most advanced T1-myelination on follow-up (Suppl.Table4).

While still clearly deficient for age, classic type PMD patients had significantly higher myelination scores than connatal and transitional patients (p < 0.001, Suppl.Table3) even if the two classic patients outside the age range of connatal and transitional patients were excluded (p = 0.003) suggesting a true difference between classic and more severely affected patients and not one due to different ages at imaging. In contrast to only early, transient T2-isointensity of PLIC in two of eight connatal and transitional patients, best T2-myelination of PLIC was at least cortex-isointense in all classic patients. MCP was hyperintense in all connatal and transitional patients, but in only three of ten classic patients (0.15, 0.21, 1.22 years) becoming T2-hypointense in the two patients with follow-up (3.15, 9.79 years). There was T1-hyperintense myelin in PLIC in all classic type PMD patients, at least T1-isointense myelin in centrum semiovale and optic radiation, and T1-hyperintense myelin in MCP excepting isointensity in one patient only imaged at 0.21 years. In contrast to T1-hypointensity of subcortical central and primary visual area in connatal and transitional patients, best myelination was at least T1-isointense in seven and eight classic patients.

While highest scores of classic patients overlapped with those of intermediate, PLP0, SPG2, and female patients, median scores in the ten patients of latter group were significantly higher (p = 0.001; Suppl. Table3). This group differed from classic, connatal, and transitional patients by absence of abnormally T2-hyperintense lemniscus with exception of one intermediate patient and presence of at least T2-isointense signal of central region in five patients, which was absent in any of the 18 classic, transitional, connatal patients. Best myelination of central region and centrum semiovale was T1-hyperintense in all patients. While scores for T1-best myelination were uniformly 11 or 12 (full score), T2-myelination was more variable with lowest scores in PLP0 (Suppl.Table3).

Vice versa, in our cohort a patient without T2-isointense myelin, without T1-hyperintensity in PLIC, T1-isointensity in optic radiation and centrum semiovale had connatal or transitional PMD. A patient with T2-hypointense myelin in MCP, at least T2-isointense myelin in centrum semiovale and PLIC, T1-hyperintense myelin in central region, centrum semiovale, optic radiation, and MCP, was either a patient with classic PMD or a milder form. If medial lemniscus was not T2-hyperintense, frontal white matter at least T2-isointense and primary visual region at least T1-isointense, the patient had a milder PMD form, namely intermediate, PLP0, SPG2, or was female (Suppl.Table4).

### 3.4. Changes of myelination score in patients with follow-up

**Fig. 4.**
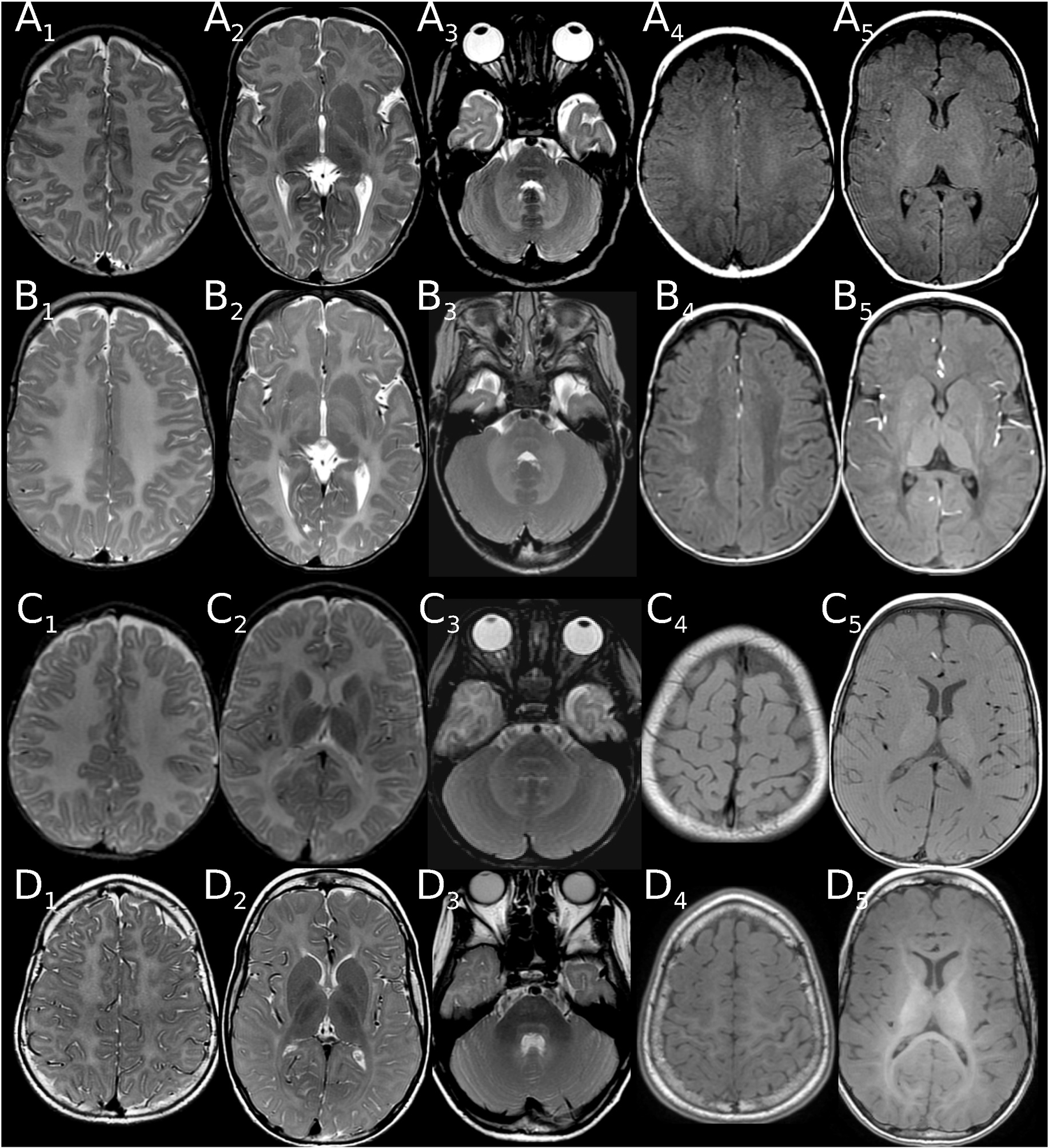
Examples of changing myelination score on follow-up. (A, B) Loss of myelin signal in a patient with connatal PMD. (A) Deficient myelination at 0.19 years with a T2-score of 1 (isointense optic radiation, A_2_; NB signal in PLIC not isointense to cortex), but near normal T1-score of 5 for age (hyperintense pyramidal tract in PLIC(A_5_), isointense in centrum semiovale (A_4_), optic radiation(A_5_), MCP (not shown)). At 0.96 years only punctate T1-isointensity of pyramidal tract in PLIC remains, while T1-isointensity in C. semiovale and T2/T1-isointensity of optic radiation (B_2,4_) has disappeared. (C, D): Progressing myelination in a patient with classic PMD. (C) Deficient myelination at 1.22 years with a T2-score of 1 (isointense pyramidal tract in PLIC (C_2_)) and a T1-score of 9 (hyperintense in PLIC, optic radiation (C_5_), MCP (not shown), isointense in central (C_4_) and prim. visual region (C_5_), centrum semiovale (not shown)). Progressing, though still severely deficient myelination at 9.79 years with a T2-score of 7 (pyr.tr. isointense in C.sem. (D_1_), hypointense in PLIC, optic radiation (D_2_), MCP (D_3_)) and a full T1-score of 12 (central region (D_4_), PLIC, optic radiation, prim. visual (D_5_), centrum semiovale; MCP (not shown)). NB T2-hyperintense medial lemniscus and pyramidal tract in pons in both patients (A-D_3_).

Follow-up MR scans for longitudinal assessment were available for 19 patients (Fig. 4, Suppl.Table2). Myelination scores changed in 13 of 19 patients (e.g. Fig. 3D–F): While still severely deficient, myelination progressed in five of seven patients with classic PMD between 0.15–1.92 and 3.15–11.42 years. Scores also increased in both intermediate-type patients and one of two female patients between 0.63–2.18 and 3.04–3.96 years. In contrast, loss of myelin signal occurred in one of four connatal and one of two transitional PMD patients between first MRI at 0.19 and 0.45 years and follow-up at 0.96, 1.62, and 4.36 years. A similarly early loss of myelin between 0.49 and 1.43 years was observed in the PLP0 patient with follow up, while it occurred after the age of five years in the SPG2 and one female patient.

### 3.5. Surrogate parameters of brain volume in PMD patients

BCR as an indicator of supratentorial volume deficit was increased to more than two standard deviations above age-normalized values (z-scores) in 13 patients, in 5 at first MRI. This was uncommon during the first year of life, present in a third of patients imaged during their second year of life and in more than half of the patients imaged after the age of two years. There was a mild, significant correlation of BRC-z-score with age for all MRIs (r = 0.282, p = 0.041). The more frequent increase of BCR in classic compared to connatal type patients (6/10 vs. 2/5; Suppl. Table 5, Suppl.Figure 4) is likely due to higher age at imaging of classic patients.

Corpus callosum was thin for age in 21 patients, in 14 at first imaging and observed earlier than increased BCR. Thin corpus callosum was present in 25% of patient imaged during the first year of life and the majority of patients imaged afterwards (Suppl.Table 5). Similar to BCR, a more commonly thin corpus callosum in classic compared to connatal type patients (9/10 vs. 2/5) is likely related to differing age at imaging. Maximal sagittal pons diameter was below −2 SDS only in one classic patient at 11.95 years and became so on follow-up at 6.6 years in one transitional patient.

## 4. Discussion

In view of evolving treatment for PMD, knowledge of the natural course and reliable biomarkers are crucial for stratification of patients and assessment of treatment effects. To this end we have developed a visual, semiquantitative PMD myelination score as a tool for standardized initial assessment and follow-up. With this score, available imaging may be used retrospectively, allowing studies on large patient cohorts who did not undergo advanced quantitative imaging studies. Using T2w and T1w axial images from routine clinical MR examinations of 28 patients we were able to differentiate PMD subtypes and document changes in myelination at follow-up. While limited by the small number of patients in subgroups and aggregation of patients based on traditional clinical phenotyping, the results suggest diverging degrees and temporal patterns of myelination in PMD subtypes, namely [1] very little myelin and early loss of myelin signal in severely affected connatal and classic patients [3], more, though still severely deficient myelin in classic PMD [4], ongoing myelination in classic and intermediate PMD during childhood [5], potential early loss in PLP0 and later loss in female and SPG2 patients. Thin corpus callosum for age and increased BCR as separately evaluated indicators of atrophy was present in 50% of patients and increased with age at imaging.

### 4.1. Subtypes and scoring of PMD

MRI patterns in PMD were first categorized by Nezu et al. as diffuse signal alteration of the cerebral hemispheres with (type 1) or without (type 2) involvement of corticospinal tracts or as (type 3) patchy signal changes. The authors found a type 1 pattern in their four patients with PLP1 duplications despite differing clinical severity and suggested that brain atrophy rather than MRI pattern (co)determined clinical severity [26]. For more precise assessment Plecko et al. not only used the MRI patterns but also scored myelination of four PMD patients in 18 regions as corresponding to, incomplete or absent for respective age [18], an approach Sarret et al. subsequently modified by evaluating 10 regions on T2w, FLAIR, and T1w images in 35 PMD patients [19]. While Plecko et al. reported a good correlation between the degree of hypomyelination and clinical severity for their four patients, Sarret et al. found no difference of myelination score between patient subgroups based on best motor acquisition, but lower scores of deep frontal white matter, ALIC, and arcuate fibres, as well as lower mean atrophy scores in the more severely affected patients with no motor achievement or only head control in their group of 35 PMD patients. In the same year Sumida et al. reported that diffuse T2-hyperintensity in brainstem and a T1-myelination age “before birth” predicted severe forms, while patchy signal alterations or a pattern of diffuse hypomyelination with some T2-hypointensity of PLIC was associated with milder phenotypes as classified by best motor acquisition. The authors modified the patterns introduced by Nezu et al. by defining the presence of some T2-hypointensity in PLIC (“best myelination) as discriminator between the two patterns of diffuse hypomyelination, changing it from involvement (“worst myelination”) of corticospinal tract to best myelination in PLIC as the specified location for assessment of corticospinal tract [27].

Comparison of these results is limited not only by differing patient numbers, clinical and MRI classifications, but also by use of involvement versus best myelination of structures and of age-normalized myelination scores versus myelination age. With the aim of establishing a myelination score for direct differentiation of PMD subgroups and monitoring on follow-up, we decided to forego age normalization as this precludes direct inter- and intraindividual comparison of values (e.g. a stable myelin deficit at 3 and 9 months resulting in different age-normalized score values) and would obscure differences in patients with less myelin than a term neonate (in our group 16/28 at first MRI for T2-items).

The score was based on axial T2w and T1w images available as part of routine clinical imaging, but not FLAIR thereby avoiding the triphasic sequence of signal changes of deep white matter on FLAIR-images as well T1-relaxation effects due to the inversion pulse used [28]. Myelination of specific structures was assessed as best myelination in order to be able to delineate partially myelinated from non-myelinated structures rather than completely from partially myelinated items. For more objective assessment the signal of specific white matter regions was scored in a semiquantitative fashion with cortical grey matter as an internal reference (except for pontine structures), and for each item examples for each value of the score were documented for training of raters.

The items included in the score were selected from a larger number scored in patients and controls based on interrater variability, practicability of scoring and importance of items for differentiation. Consequently, the score as a model of myelination does neither reproduce the entire process of myelination nor sample all lobes and white matter structures as earlier myelination scores for PMD and 4H have done [16, 18,19] but was tailored to PMD. As a result, it is “skewed” towards early myelinating, reliably discernible structures found to be central for differentiation of PMD and its subgroups in our cohort of 28 patients. Skewing toward early myelination and the extent of hypomyelination encountered in PMD is underscored by the fact that controls attain full scores between 3.5 and 4.6 months for T1-items, between 8.7 and 10.8 months for T2-items and thus also for the resulting myelination score of the combined T2-and T1-items. While this disease-orientated score development allowed us to include as few items as possible, it makes validation in a larger cohort necessary. Nevertheless, reviewing the literature on MRI changes reported in PMD there is some support for the items upon which the proposed myelination score is built: Consistent with previous studies, T2-hyperintense PLIC was only found in severely affected patients with connatal or transitional PMD [27,29–31]. Sumida et al. reported T1-myelination not equivalent to birth - defined by lack of T1-hyperintensity in PLIC and optic radiation - as predictive for severe forms of PMD [27], a finding reproduced in our patients in whom T1-hypointense PLIC and optic radiation were only found in connatal or transitional PMD. In the largest study of myelination reported [19], the age-normalized myelination of arcuate fibers as well as of frontal white matter and ALIC was less in severely affected patients without motor acquisition or only achieving head control [19]. While we discarded ALIC due to larger interrater variability, which might be due to the craniocaudal temporospatial gradient of myelination in ALIC we observed in our controls, lobar white matter in centrum semiovale and myelination of subcortical white matter also differed between subgroups of our patients: At least T1-isointense myelin of central and primary visual subcortical white matter and T2-isointense myelin in centrum semiovale was only observed in classic and milder forms of PMD, and at least T2-isointense myelin in subcortical Rolandic and frontal white matter was only present in PMD subtypes milder than classic. Interestingly, subcortical white matter of primary visual region was T2-hyperintense in all our patients, even if myelin was visible in the later myelinating subcortical frontal (not Rolandic) white matter, which occurred in two intermediate, one PLP0, one SPG2, and one female patients.

Brainstem involvement in form of patchy myelination not further specified has been reported in patients with connatal PMD [31,32] and symmetric T2-hyperintensity of pontine pyramidal tract in classic patients [26]. While we did not include T2-hyperintense pontine pyramidal tract due to lower interrater variability and difficulty of scoring with overlaying artefacts in some patients, it was a common finding present in 19 of our 28 patients (5/5 connatal, 3/3 transitional, 9/10 classic and 1/3 intermediate including secondary T2-hyperintensity at FU in 2 classic and 1 intermediate patients, 1/3 PLP0, 0/3 SPG2, 1/2 female). The finding of T2-hyperintense medial lemniscus was just as common, but more restricted (all connatal, transitional, classic, 1/3 intermediate patients), T2-hyperintensity of MCP less common and even more restricted (all connatal, transitional, 3/10 classic patients, 2/3 with mature myelin on FU). The identical structures are depicted in the example of diffuse brainstem hyperintensity, which Sumida et al. only found in severe PMD without motor acquisition, whereas partial hyperintensity with only pyramidal tract hyperintensity depicted in the example was found even in SPG patients (Figs. 1B and 2B in Ref. [27]), which is similar to our findings.

### 4.2. Temporal patterns and determinants of clinical disability in PMD

Previous studies in larger groups of patients have found that white matter atrophy is a major determinant of clinical disability [17,19,27], while extent of hypomyelination at least codetermines motor handicap in PMD. Despite handicap, children with PMD commonly develop to reach a plateau at about 10 years and later decline. Ongoing, though delayed and still severely deficient myelination is one potential correlate. This occurred in five of our seven classic, both intermediate and one of two female patients between first imaging in infancy and follow-up at 3–12 years (5/7 classic, 2/2 intermediate, 1/2 female) and has previously been documented in patients who achieved at least sitting position, namely two patients first imaged around one year of age at follow-up at 2–5 years and in seven patients between three and 12 years [18,19,27]. On the other hand, some patients lose myelin signal. Early loss during infancy was present in our two connatal and transitional patients with the least deficient myelination who were also among the youngest imaged. Early loss of myelin however does not seem to be exclusive to the most severe forms, occurring also in one of our PLP0 patients and a previously reported classic patient (between 3.5 and 6 months in case 2 in Ref. [30]). Later loss has been observed in a juvenile patient [19] as well as in late childhood in one of our female and the SPG2 patient with follow-up and may contribute to decline in patients. Atrophy however seems a much more likely candidate as a biomarker for decline, correlating with functional disability and increasing with age. Volume loss in our patients began in the second year of life and thus earlier than the five to seven years reported by Sarret et al. for their group which might be due different age distributions in patient groups: Reviewing the graphs only two of 17 patients with follow-up in Sarret et al. were imaged before the age of five years, whereas 17 of our 19 patients with follow-up had at least one MRI before the age of 5 years and 14 of 19 before the age of 2 years. Contrary to expectation, atrophy scores in our patients were less in connatal than classic patients which most likely resulted from earlier imaging of connatal patients. Nevertheless, this finding illustrates, that combination of myelination score and atrophy into one MRI score may be counterproductive for initial stratification of patients, as lower brain volume scores but higher myelination scores of classic compared to connatal type PMD patients resulted in blurring of score boundaries.

To summarize, the proposed PMD myelination score is based on routine MR images, differentiates clinical PMD subtypes, detects and quantifies changes on follow-up. The score needs to be validated in a larger cohort with subgroups based on best motor function instead of onset and rate of progression of the traditional clinical phenotyping as do surrogate parameters of brain volume as markers of secondary degeneration for a more comprehensive, integrated MRI score for PMD. This will also aid in understanding differences in natural history, i.e. some progression of myelination in classic and intermediate PMD and loss in severely affected patients and PLP0, which need to be taken into account when assessing efficacy in treatment trials.

## Supplementary Material

supplement

## Figures and Tables

**Table 1:** Brain myelination score for PMD.

PMD myel. score	item	reference	T2w	T1w

pyramidal tract	central region^a^	cortex	0–2	0–2
	centrum semiovale^a^	cortex	0–2	0–2
	PLIC	cortex	0–2	0–2
visual tract	optic radiation	cortex	0–2	0–2
	primary visual cortex	cortex	0–2	0–2
other	MCP	cortex	0–2	0–2
	frontal (not central)	cortex	0–2	–
	medial lemniscus	surrounding	0–1	–
sum			0–15	0–12
myel. score			0–27	

Score values: 0 = T2-hyper/T1-hypointense, 1 = T2/T1-isointense, 2 = T2-hypo/T1-hyperintense relative to cortex except for medial lemniscus (0 = T2-hyperintese relative to surrounding, 1 = T2-iso/hypointense).

Abbreviations: MCP: middle cerebellar peduncle; PLIC: posterior limb of internal capsule; T2w/T1w: T2/T1-weighted.

a Compare with cortex of central region as this is more T2-hypointense/T1-hyperintense than adjacent cortex (Figs. 1 and 2 A, B).
