# Supplementary material for: Identification of PMD subgroups using a myelination score for PMD
Source: Eur J Paediatr Neurol. Author manuscript; Available in PMC 2024 Aug 27. (PMC11348679; doi:10.1016/j.ejpn.2022.10.003)

Suppl. Material, Suppl.Tables 1-4, Suppl.Figures 1-3

Supplementary Material: Selection of T2- and T1-items for the myelination score

T2-items

Visually, a score consisting of the five supratentorial T2-items of pyramidal and visual tract, namely central region, centrum semiovale, PLIC, optic radiation, and primary visual area, was sufficient to differentiate PMD patients and controls. Inclusion of additional T2-items did not change discrimination between PMD patients and controls either visually or on ROC analysis for different T2-scores with AUC between 93.9% and 94.2% (Suppl.Fig.3). Additional items, however, were helpful in differentiating PMD subgroups: With inclusion of medial lemniscus, MCP, and frontal subcortical white matter, AUC increased from 90.0% to 96.2% for differentiation of connatal and transitional from classic PMD and from 86.0% to 97.0% for differentiation of classic versus milder forms of PMD (intermediate, PLP0, SPG2, female;). Subcortical parieto-occipital, peridentate white matter, white matter in foliae and corpus callosum were discarded as inclusion only increased AUC up to 98.3% but at the cost of more difficult scoring. The resulting T2-score consisted of 8 items, namely supratentorial pyramidal (n=3) and visual tract (n=2), subcortical frontal white matter, medial lemniscus, and MCP, with a maximum score value of 15 and cut-off values of 0.5 and 3 for differentiation of classic vs. connatal and transitional and classic vs. milder forms, respectively (Table 1, Figure 3A, Suppl.Figure 3A).

T1-items:

T1-items were helpful for discriminating patient subgroups but were not primarily suited for differentiating patients and controls due to more extensive myelin signal on T1w images with overlap between patients and controls. A score consisting of the five T1-items of supratentorial pyramidal and visual tract had an AUC of 73.9% for separation of patients and controls, of 98.1% for differentiation of classic from connatal and transitional PMD and of 85.5% for classic vs. milder PMD forms. ALIC, genu, and splenium were discarded due to difficulty or relatively low

concordance of scoring. Subcortical frontal and parietal white matter (not central, not cuneus) were discarded as they did not separate patient subgroups. Addition of MCP resulted in 100% separation for classic vs. connatal and transitional PMD, while separation of classic vs. milder PMD remained at 85.5%. While peridentate white matter and white matter of foliae also differentiated subgroups, they did not further increase AUC, but were more difficult to score and therefore discarded. Consequently, the six T1-items of supratentorial pyramidal (n=3) and visual tract (n=2) and MCP were retained for the score with a maximal value of 12 and cut-off values of 5.5 and 10 for differentiation of classic vs. connatal and transitional and classic vs. milder forms (Table 1, Figure 3B, Suppl.Figure 3B).

Suppl.Table 1: List of T2- and T1-items scored.

Score values: 0 = T2-hyper/T1-hypointense, 1 = T2/T1-isointense, 2 = T2-hypo/T1-hyperintense relative to cortex except for medial lemniscus (0 = T2-hyperintese, 1 = T2-iso/hypointense relative to surrounding). Abbreviations: ALIC: anterior limb of internal capsule; MCP: middle cerebellar peduncle; PLIC: posterior limb of internal capsule; T2w/T1w: T2/T1-weighted; wm: white matter; *compare with cortex of central region as this is more T2-hypointense than adjacent cortex (Fig. 2A, B); **simplified scoring as 0 (T2-hyper/T1-hypoint.) vs. 1 (iso/T2-hypo/T1-hyperint.); ***not scored on T1w images.

	item	wm region	reference	value
pyramidal tract	central region*	subcortical	cortex*	0-2
	centrum semiovale*	deep	cortex*	0-2
	PLIC	int. capsule	cortex	0-2
visual tract	optic radiation	periventricular	cortex	0-2
	primary visual cortex	subcortical	cortex	0-2
corpus callosum	splenium	central	cortex**	0-1
	genu	central	cortex**	0-1
other supratentorial	ALIC	int. capsule	cortex	0-2
	frontal (not central)	subcortical	cortex	0-2
	parieto-occipital (not central, cuneus)	subcortical	cortex	0-2
brainstem & cerebellum	pyramidal tract in pons	pons	surrounding***	0-1
	medial lemniscus	pons	surrounding***	0-1
	MCP	pons	cortex	0-2
	peridentate white matter	deep	cortex	0-2
	foliae (caudal hemispheres, not flocculonodular lobe)	subcortical	cortex	0-2

Supp.Table 2: Overview of patients, subgroups, and age at MRI.

		n pat. (n with FU)	n MRI	age (yrs; FU0 / all MRIs) range	mean	median
all patients		28 (19)	53	0.15-11.95 / 0.15-14.42	2.85 / 4.07	1.30 / 2.18
subgroups	connatal	5 (4)	9	0.17-0.46 / 0.17-2.64	0.29 / 0.76	0.22 / 0.46
	transitional	3 (2)	6	0.45-1.91 / 0.45-6.60	1.04 / 2.62	0.76/ 1.77
	classic	10 (7)	19	0.15-11.95 / 0.15-11.95	2.59 / 4.70	1.30 / 3.15
	intermediate	3 (2)	6	0.63-2.18 / 0.63-7.15	1.45 / 3.14	1.53 / 2.61
	PLP0	3 (1)	4	0.49-11.72 / 0.49-11.72	7.63 / 6.08	10.7 / 6.05
	SPG2	2 (1)	4	4.07-10.20 / 4.07-10.20	7.14 / 6.39	7.14 / 5.64
	female	2 (2)	5	2.03-5.88 / 2.03-14.42	3.96 / 6.98	3.96 / 5.88

Suppl.Table 3: Myelination scores in PMD subgroups at first MRI and for all MRI scans.

**FU0** subgroups	n pat.	age (yrs) range FU0	scores (mean / median / [range]) T2	T1	T21	cut-offs (ROC) T2 / T1 / T21
connatal	5 (4)	0.08-0.41	0.2 / 0 / [0-1]	1.8 / 2 / [0-5]	2.0 / 2 / [0-6]	conn&trans vs. classic:
transitional	3 (2)	0.45-1.91	0.3 / 0 / [0-1]	2.67 / 3/ [1-4]	3.0 / 3 / [1-5]	0.5 / 5.5 / 6.5
classic	10 (7)	0.15-11.95	2.1 / 2 / [1-4]	8.7 / 9 / [6-12]	10.8 / 10.5 / [7-16]	
intermediate	3 (2)	0.63-2.18	6.3 / 7 / [5-7]	11.7 / 12 / [11-12]	18.0 / 19 / [16-19]	classic vs.
PLP0	3 (1)	0.49-11.72	5.0 / 4 / [4-7]	11.7 / 12 / [11-12]	16.7 / 16 / [15-19]	interm&PLP0&SPG&fem:
SPG2	2 (1)	4.07-10.20	7.5 / 8 / [4, 11]	11.5 / 11.5 / [11, 12]	19.0 / 19 / [15, 23]	3 / 10 / 14.5
female	2 (2)	2.03-5.88	8.0 / 8 / [7, 9]	12.0 / 12 / [12, 12]	20.0 / 20 / [19, 21]	

**all MRIs** subgroups	n MRI	age (yrs) range all	scores (mean (range)) T2	T1	T21	cut-offs (ROC) T2 / T1 / T21
connatal	9	0.08-2.64	0.1 / 0 / [0-1]	1.7 / 2 / [0-5]	1.8 / 2 / [0-6]	conn&trans vs. classic:
transitional	6	0.45-6.60	0.2 / 0 / [0-1]	2.0 / 1.5 / [1-4]	2.2 / 1.5 / [1-5]	0.5 / 5.5 / 6.5
classic	19	0.15-12.0	3.4 / 4 / [1-7]	10.2 / 11 / [6-12]	13.5 / 15 / [7-19]	
intermediate	6	0.63-7.51	8.0 / 8 / [5-10]	11.8 / 12 / [11-12]	19.8 / 20 / [16-22]	classic vs.
PLP0	4	0.49-11.7	4.5 / 4 / [3-7]	11.5 /11.5 / [11-12]	16.0 / 15.5 / [14-19]	interm&PLP0&SPG&fem:
SPG2	4	4.07-10.2	9 / 10.5 / [4-11]	11.8 / 12 / [11-12]	20.8 / 22.5 / [15-23]	6.5 / 10 / 18.5
female	5	2.03-5.88	7 / 7 / [3-11]	12 / 12 / [12]	19.0 / 19 / [15-23]	

Suppl.Table 4: Range of scores for T2- and T1-items in PMD subtypes.

Score values: 0 = T2-hyper/T1-hypointense, 1 = T2/T1-isointense, 2 = T2-hypo/T1-hyperintense. Abbreviations: central: subcortical white matter of central region; C.sem.: centrum semiovale, front.: subcortical frontal white matter, not central; MCP: middle cerebellar peduncle; lemn.med.: medial lemniscus; opt.rad.: optic radiation; prim.vis: subcortical white matter of primary visual cortex; trans.: transitional.

T2 (all MRIs)	pt.	central	C.sem.	PLIC	opt.rad.	prim.vis.	MCP	lemn.med.	front.
connatal	5	0	0	0	0 (1 trans.)	0	0	0	0
transitional	3	0	0	0 (1 trans.)	0	0	0	0	0
classic	10	0	0 (-1*)	1 -2	0-1 (-2*)	0	0-2	0	0
intermediate	3	0-1 (-2*)	0-1	2	1	0	1 -2	0-1	0-2
PLP0	3	0-1	0	1	0 (1 trans.)	0	1 -2	1	0-2
SPG2	2	0-2	0-2	1 -2	0	0	2	1	0-2
female	2	0-2	0-1	(1*-) 0-2	0-1	0	2	1	0-2
		*in 1 at FU	*in 2 at FU	*1 loss at FU	*in 1 at FU				
		if 1 intermediate or better	if 1 classic or better	if 1 classic or better			if 2 classic or better	if 1 intermediate or better	if 1 intermediate or better

T1 (all MRIs)	pt.	central	C.sem.	PLIC	opt.rad.	prim.vis.	MCP
connatal	5	0	0 (1 trans.)	0-1 (trans. 2)	0 (1 trans.)	0	0-1
transitional	3	0	0	0-2	0 (1 trans.)	0	1
classic	10	0-2	1 – 2	2	1 -2	0-2	(1*-) 2
intermediate	3	2	2	2	2	1- 2	2
PLP0	3	2	2	2	1 -2	1 -2	2
SPG2	2	2	2	2	1 -2	1 -2	2
female	2	2	2	2	2	2	2
							*1 at 0.21 yrs.
		if 2 classic or better	if 2 classic or better		if 2 classic or better	if 1 classic or better	if 2 classic or better

Suppl.Table 5: BCR and thin corpus callosum in PMD subgroups.

**all MRIs** subgroups	n pat.	age (yrs) range	all ages ↑**BCR.z > 2**	0-12 mo	12-24 mo	> 24 mo
connatal	5	0.08-0.41	2/5	1/5	0/1	1/1
transitional	3	0.45-1.91	2/3	0/2	0/2	2/2
classic	10	0.15-11.95	6/10	1/3	2/5	5/8
intermediate	3	0.63-2.18	1/3	0/1	0/1	1/2
PLP0	3	0.49-11.72	1/3	0/1	1/1	0/2
SPG2	2	4.07-10.20	0/2	-	-	0/2
female	2	2.03-5.88	1/2	-	-	1/2
			13	2/12	3/10	10/19
			**CC thin**			
connatal	5	0.08-0.41	2/5	1/5	0/1	1/1
transitional	3	0.45-1.91	3/3	1/2	2/2	2/2
classic	10	0.15-11.95	9/10	1/3	5/5	8/8
intermediate	3	0.63-2.18	2/3	0/1	1/1	1/2
PLP0	3	0.49-11.72	2/3	0/1	1/1	1/2
SPG2	2	4.07-10.20	1/2	-	-	1/2
female	2	2.03-5.88	2/2	-	-	2/2
			21	3/12	9/10	16/19

Suppl.Figure 1: Age-related sampling of control MRIs for assessment of normal myelination.

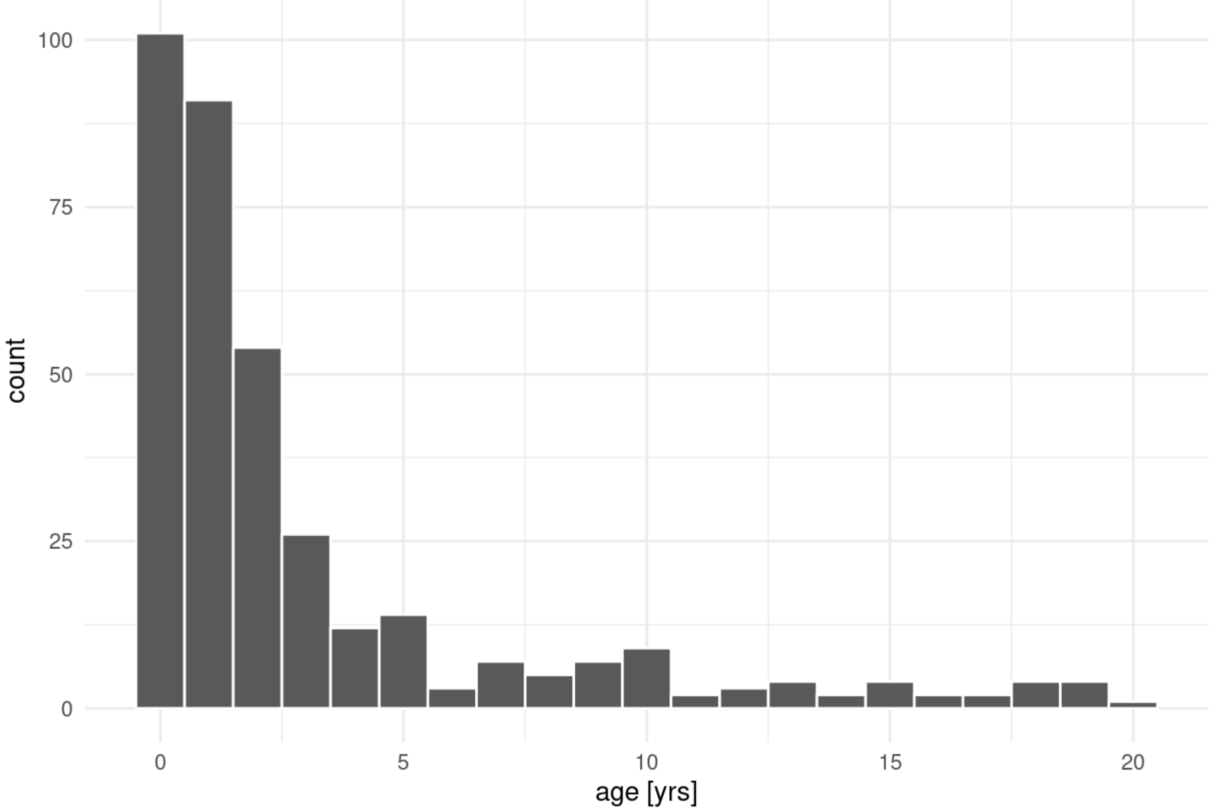

Suppl.Figure 2: Visual scoring of corpus callosum (CC) on midsagittal images.
CC is normal for age in a patient with connatal PMD at 2.6 months (A), unchanged and now thin for age on follow-up at 7.4 months (B; youngest patient with visual scoring of thin CC for age). Compare with normal CC for age in a classic patient at 7.6 months (C) and a control patient at 7.4 months (D).

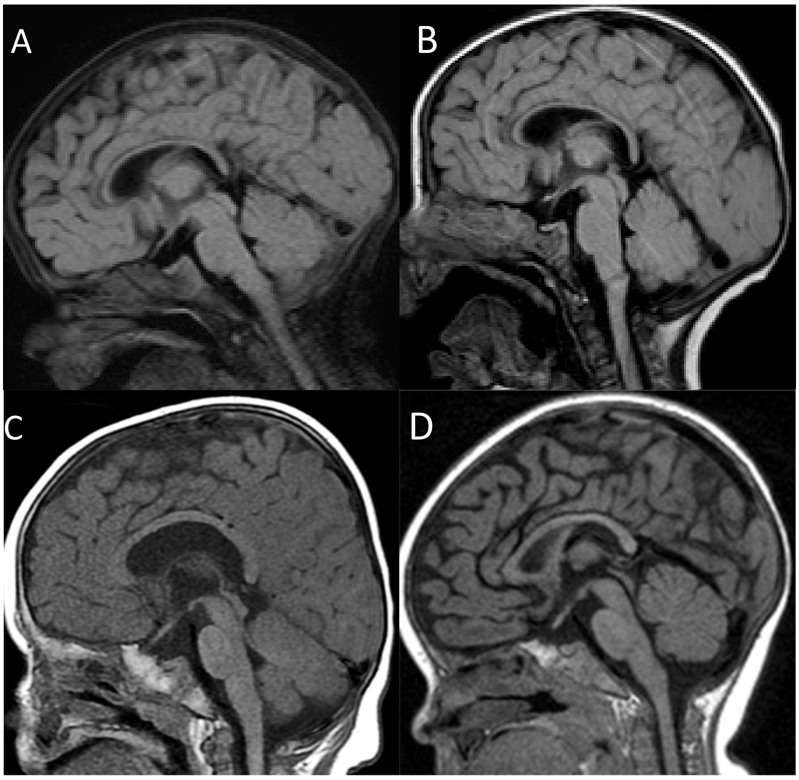

Suppl.Figure 3A: Receiver operating characteristics (ROC) of T2- (A), T1- (B), and T21-scores (C) for differentiation of PMD and controls and of PMD subgroups. ROCs are depicted for basic scores consisting of supratentorial pyramidal and visual tract and additional items (“pyr_opt”; central region, C. semiovale, PLIC, optic radiation, prim. visual area) and final scores with additional items for better subgroup differentiation (T2: medial lemniscus, MCP, front.white matter, T1: MCP, T21: respective additional T2- and T1- items).

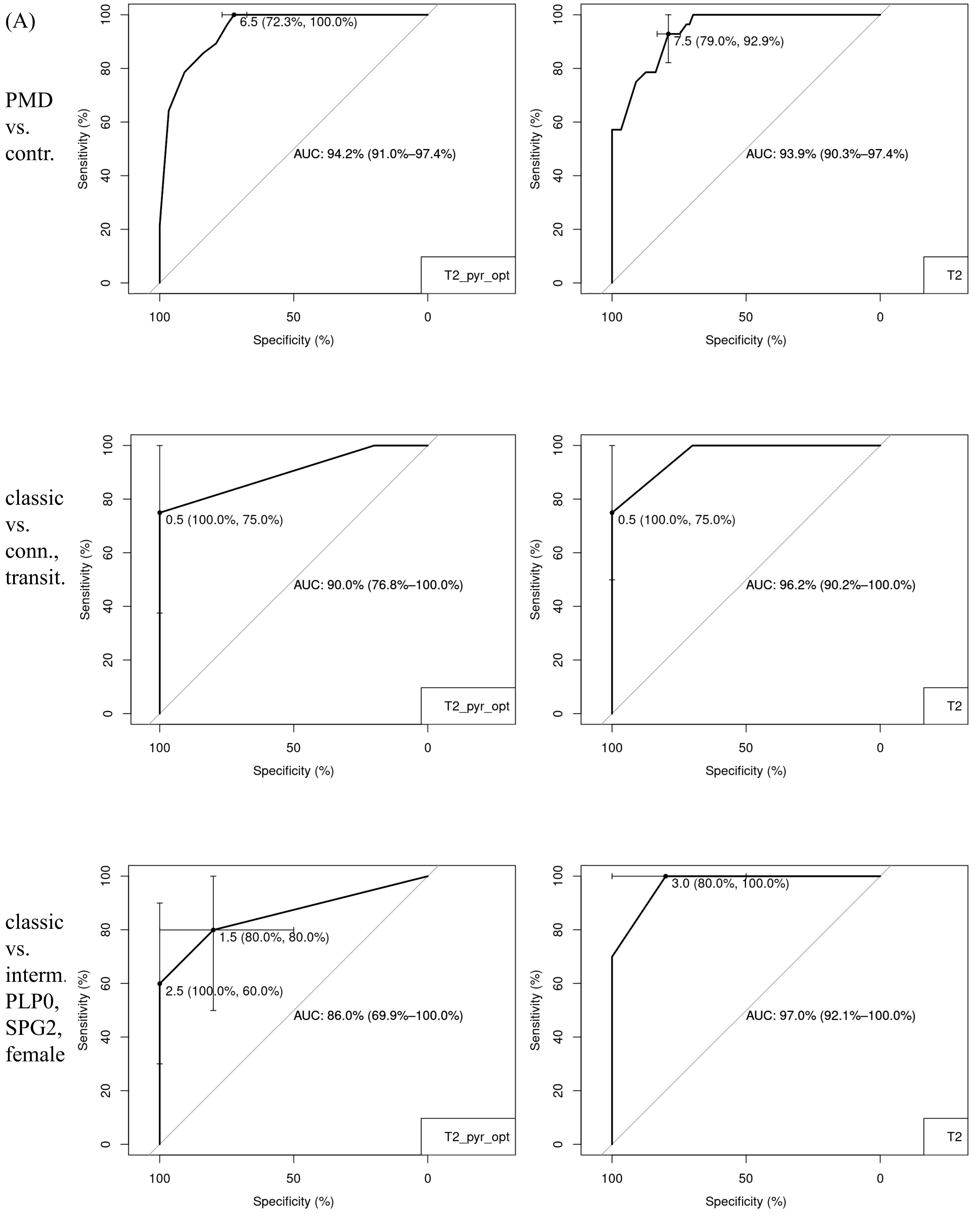

<u>Suppl.Figure 3B</u>: Receiver operating characteristics (ROC) of T2- (A), T1- (B), and T21-scores (C) for differentiation of PMD and controls and of PMD subgroups. ROCs are depicted for basic scores consisting of supratentorial pyramidal and visual tract and additional items ("pyr_opt."; central region, C. semiovale, PLIC, optic radiation, prim. visual area) and final scores with additional items for better subgroup differentiation (T2: medial lemniscus, MCP, front.white matter, T1: MCP, T21: respective additional T2- and T1- items).

(B)

PMD vs. contr.

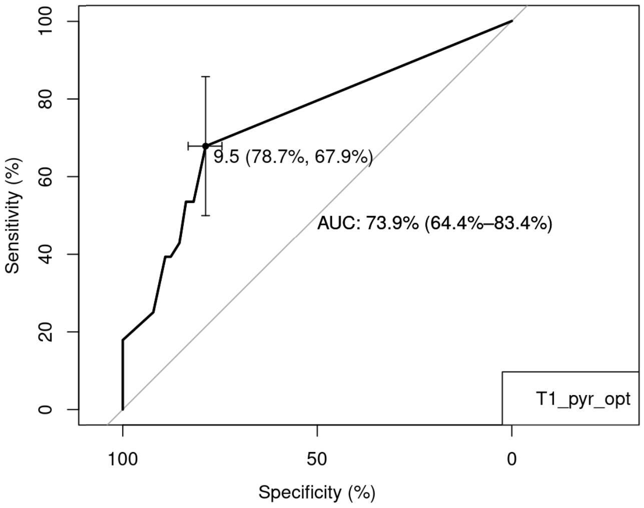

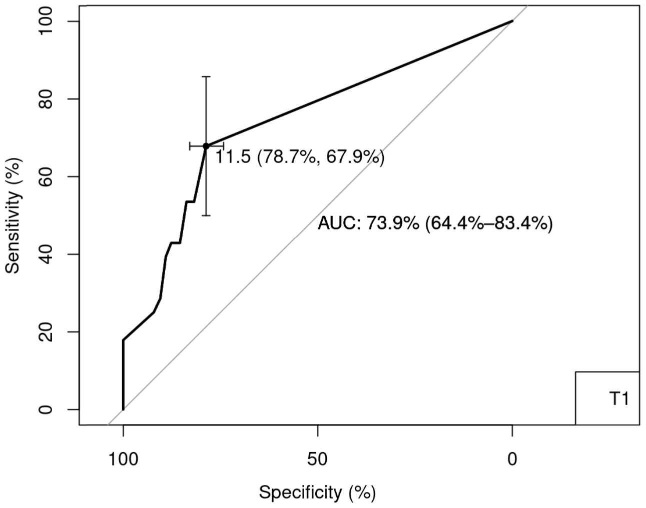

classic vs. conn., transit.

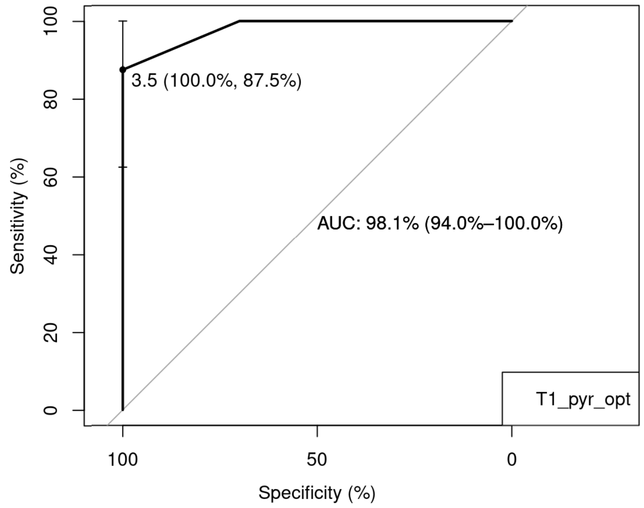

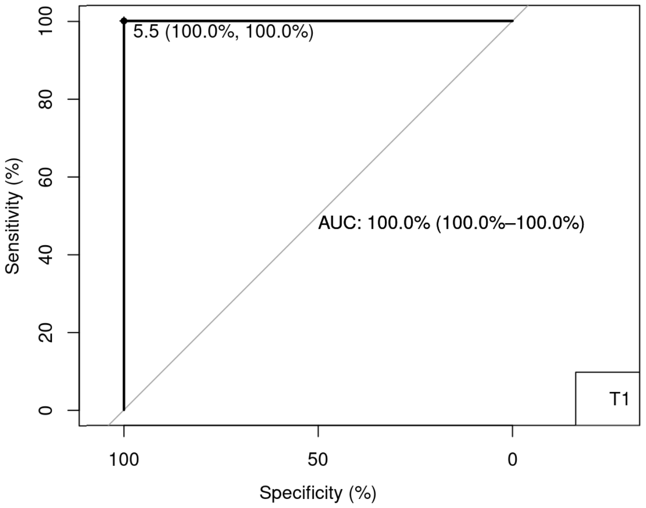

classic vs. interm PLP0, SPG2, female

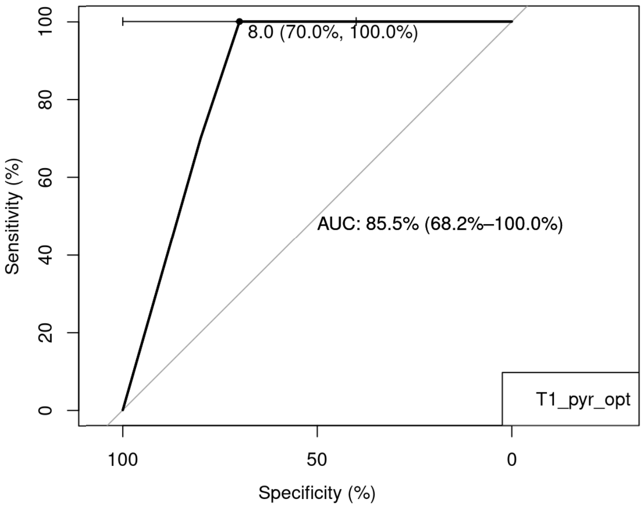

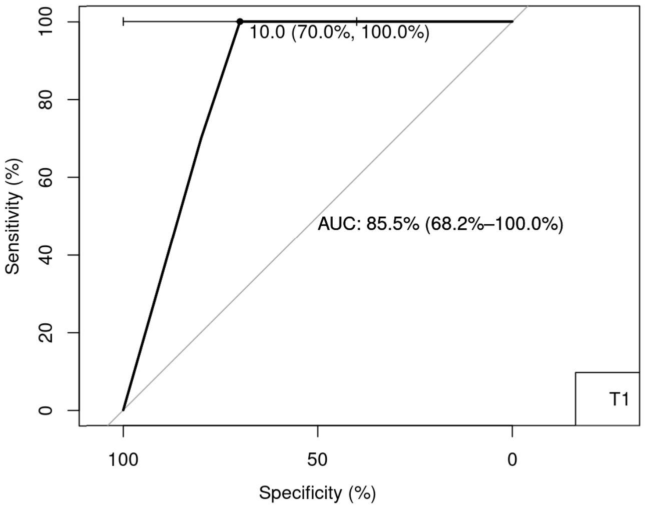

<u>Suppl.Figure 3C</u>: Receiver operating characteristics (ROC) of T2- (A), T1- (B), and T21-scores (C) for differentiation of PMD and controls and of PMD subgroups. ROCs are depicted for basic scores consisting of supratentorial pyramidal and visual tract and additional items (“pyr_opt.”; central region, C. semiovale, PLIC, optic radiation, prim. visual area) and final scores with additional items for better subgroup differentiation (T2: medial lemniscus, MCP, front.white matter, T1: MCP, T21: respective additional T2- and T1- items).

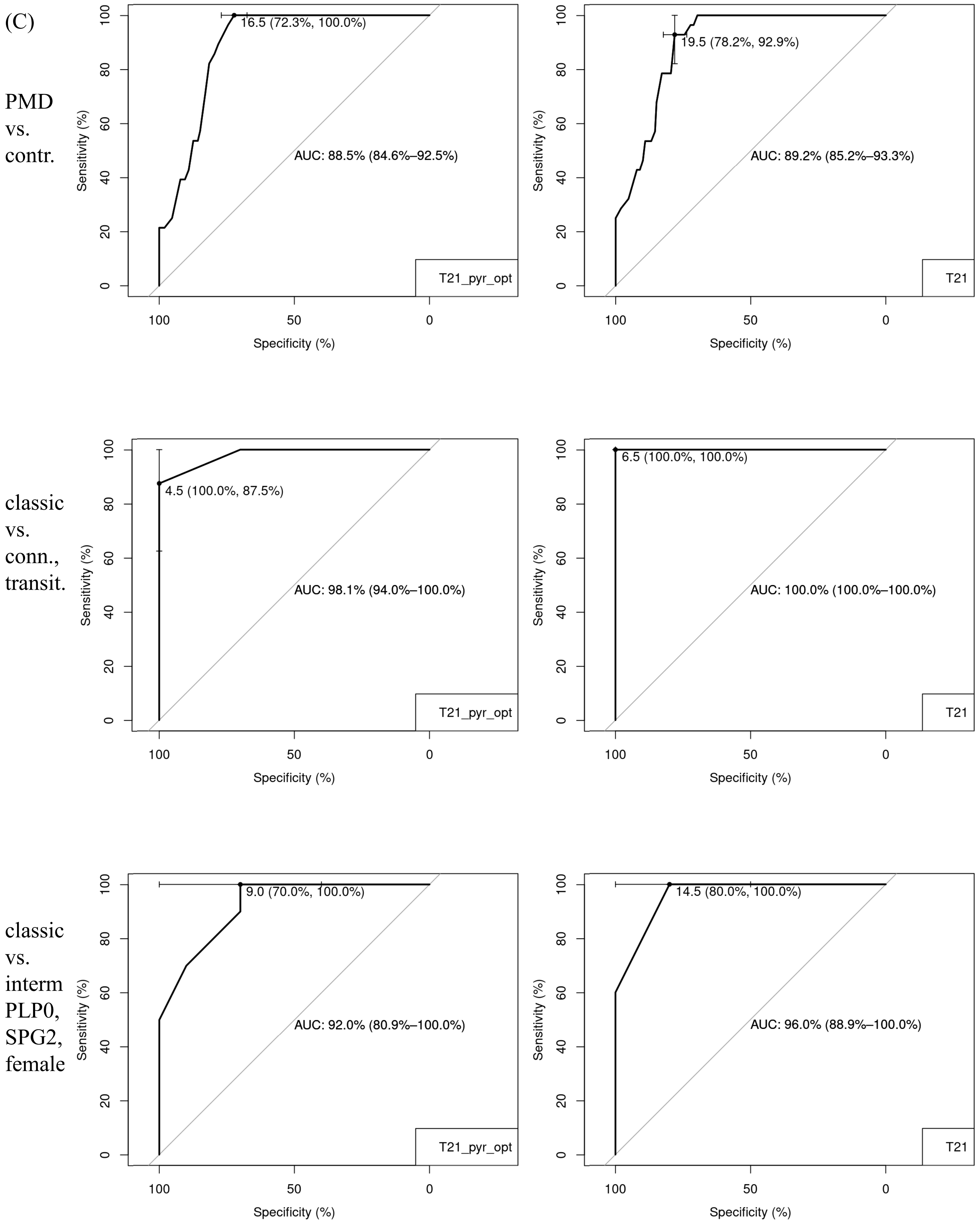

Suppl.Figure 4: BCR-z-scores across age for all MRI scans of PMD patients.

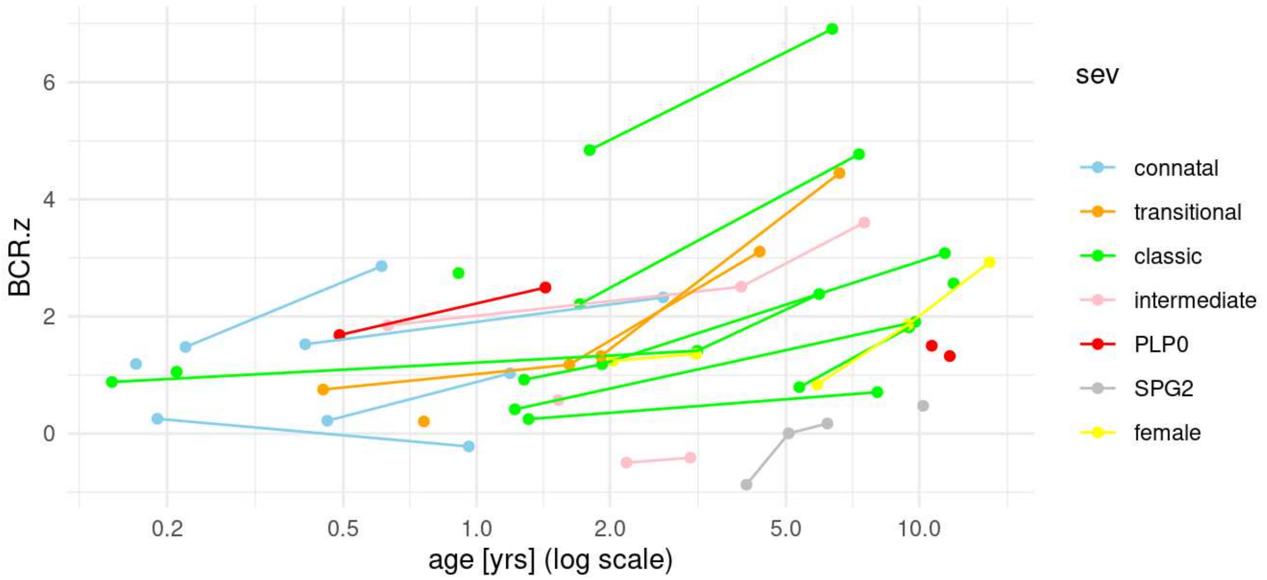

Supplement: supplement [file NIHMS2014627-supplement-supplement.pdf]
